# Pharmacological targeting of the hyper-inflammatory response to SARS-CoV-2-infected K18-hACE2 mice using a cluster of differentiation 36 receptor modulator

**DOI:** 10.3389/fphar.2024.1303342

**Published:** 2024-02-07

**Authors:** Jade Gauvin, David N. Huynh, Isabelle Dubuc, Catherine Lê, Rafaela Tugores, Nicolas Flamand, Louis Flamand, William D. Lubell, Huy Ong, Sylvie Marleau

**Affiliations:** ^1^ Faculty of Pharmacy, Université de Montréal, Montréal, QC, Canada; ^2^ Department of Microbiology, Infectious Diseases and and Immunology, Université Laval, Québec, QC, Canada; ^3^ Institut Universitaire de Cardiologie et de Pneumologie de Québec, Département de Médecine, Faculté de Médecine, Université Laval, Québec, QC, Canada; ^4^ Department of Chemistry, Université de Montréal, Montréal, QC, Canada

**Keywords:** coronavirus, SARS-CoV-2, hyper-inflammation, cytokine storm, pneumonia, macrophages, azapeptides, CD36

## Abstract

The scientific and medical community faced an unprecedented global health hazard that led to nearly 7 million deaths attributable to the rapid spread of severe acute respiratory syndrome coronavirus 2 (SARS-CoV-2) infection. In spite of the development of efficient vaccines against SARS-CoV-2, many people remain at risk of developing severe symptoms as the virus continues to spread without beneficial patient therapy. The hyper-inflammatory response to SARS-CoV-2 infection progressing to acute respiratory distress syndrome remains an unmet medical need for improving patient care. The viral infection stimulates alveolar macrophages to adopt an inflammatory phenotype regulated, at least in part, by the cluster of differentiation 36 receptor (CD36) to produce unrestrained inflammatory cytokine secretions. We suggest herein that the modulation of the macrophage response using the synthetic CD36 ligand hexarelin offers potential as therapy for halting respiratory failure in SARS-CoV-2-infected patients.

## Introduction

Severe acute respiratory syndrome coronavirus 2 (SARS-CoV-2) pandemic caused over 773 million infections and nearly 7 million deaths globally (WHO, 2023) between 2019 and 2023. Most infected patients were either asymptomatic or manifested mild to moderate flu-like symptoms common among viral respiratory tract infections. Some individuals, notably the elderly and those presenting comorbidities, often developed a rapid deterioration of respiratory functions and lower airway infection progressing to acute respiratory distress syndrome (ARDS) and in many instances, death (Ramadori, 2022; Zheng et al., 2022). In severe cases, COVID-19 (coronavirus disease 2019)-related ARDS has been typically associated with hyper-cytokinemia, so-called “cytokine storm,” featuring macrophage activation (Tao et al., 2023). Hyper-cytokinemia is initiated by different macrophage subsets in the lungs including interstitial, alveolar, and monocyte-derived macrophages, in particular (Merad and Martin, 2020; Gustine and Jones, 2021), which contribute to ARDS development in SARS-CoV-2-infected patients (Kosyreva et al., 2021).

The cluster of differentiation 36 receptor (CD36) is an extensively glycosylated class B2 scavenger receptor, that sits at the crossroad between lipid metabolism and innate immune response. Largely expressed in immune and non-immune cells, CD36 plays important immunomodulatory roles in health and disease (Silverstein and Febbraio, 2009). First identified as a long chain fatty acid transporter, CD36 has been later shown to mediate inflammatory signaling in response to damage-associated molecular pattern signals (DAMPs), such as oxidized phospholipids (oxPLs) (Miller et al., 2011; Chen et al., 2022; Dunigan-Russell et al., 2023). The binding domain for oxPLs has been identified in the CD36 protein (Ashraf et al., 2009). As a co-receptor of Toll-like receptor (TLR) heterodimer complexes TLR2/6 (Mellal et al., 2019) and TLR4/6 (Stewart et al., 2010), CD36 is implicated in triggering nuclear factor-kappa B (NF-κB) inflammatory signaling following exposure to oxPLs (Park, 2014). Moreover, CD36 was found to modulate pro-inflammatory macrophage phenotype (Sun et al., 2022). Appropriately, the pulmonary hyper-inflammation associated with SARS-CoV-2 infection was shown to rely, in part, upon the stimulation of the pro-inflammatory NF-κB signaling pathway by oxPL accumulation in the lungs and activation of monocyte-derived macrophage TLRs (Merad and Martin, 2020). Furthermore, CD36 was found to bind SARS-CoV-2-E (envelope) protein, which is a major viral structural protein having central roles in cytokine secretion, progression to ARDS-like symptoms and thrombosis in mice (Tang et al., 2023). Accordingly, CD36 has been proposed as target for severe COVID-19 patients (Vlasov et al., 2021; Alghanim et al., 2022).

Ligands of CD36 derived from growth hormone-releasing peptides (GHRPs) such as hexarelin [H-His-2-methyl-D-Trp-Ala-Trp-D-Phe-Lys-NH_2_] and azapeptide analogs were reported to exhibit high binding affinity to CD36 in murine cardiac membranes (Bodart et al., 2002) and to exert anti-inflammatory effects (Bodart et al., 2002; Bessi et al., 2012; Huynh et al., 2017; Proulx et al., 2020). For example, azapeptide MPE-001 [H-His-D-Trp-Ala-azaTyr-D-Phe-Lys-NH_2_] was shown to palliate inflammation in response to photo-oxidative stress in mice by attenuating NF-κB and NOD-like receptor family, pyrin domain containing 3 (NLRP3) inflammasome pathways and concomitantly, by activating peroxisome proliferator-activated receptor gamma coactivator 1-alpha (PPAR-γ-PGC1α) signaling, to promote macrophage polarization towards an anti-inflammatory phenotype (Mellal et al., 2019). Various cellular responses to stimuli have been modulated by such peptide-based CD36 ligands (Proulx et al., 2020). For example, hexarelin has demonstrated cardioprotective effects in rodent models of cardiovascular dysfunction after daily subcutaneous administrations for up to 5 weeks (Ghigo et al., 1999; Locatelli et al., 1999). Cardiotropic activity has also been observed after acute administration of hexarelin to normal and growth hormone-deficient humans as well as during by-pass surgery (Bisi et al., 1999; Ghigo et al., 1999; Mao et al., 2014). In this perspective, evidence is provided to suggest that the pharmacologic modulation of CD36 response could attenuate the macrophage-driven hyper-inflammatory response observed in SARS-CoV-2-infected patients.

### Pro-inflammatory macrophage phenotype and COVID-19

Activated macrophages release cytokines [e.g., interleukin (IL)-1β, IL-6, and tumor necrosis factor (TNF)-α] and chemokines [e.g., C-X-C ligand 8 (CXCL8) and C-C ligand 2 (CCL2)] (Atri et al., 2018; Knoll et al., 2021). Cytokines and chemokines recruit leukocytes to the lungs, leading to injury of endothelial and epithelial tissues with heightened inflammation in COVID-19 patients (Ragab et al., 2020). Moreover, COVID-19 induces endothelial and epithelial alveolar damage coupled to enhanced interstitial and alveolar permeability to proteins and fluids, reduced endothelial nitric oxide, and increased reactive oxygen species (ROS) production (Chernyak et al., 2020; Yuan et al., 2021; Otifi and Adiga, 2022). Prevention of the cytokine storm in SARS-CoV-2-infected patients remains relevant and of high priority (Kosyreva et al., 2021).

## Current therapies for SARS-CoV-2-infected patients

Vaccination programs against SARS-CoV-2 have been effective but have suffered from inequality in global distribution and administration (Bayati et al., 2022). Furthermore, vaccination rates against COVID-19 are decreasing globally (Mathieu et al., 2020). In spite an apparent decline in viral burden, the risk of future coronavirus outbreaks is a major healthcare concern (Cui et al., 2023). Current COVID-19 therapeutics function typically as antiviral and anti-inflammatory agents (Niknam et al., 2022; Yuan et al., 2023). For example, patients presenting mild to moderate COVID-19 symptoms are treated with Paxlovid, which features the viral 3C-like protease inhibitor nirmatrelvir boosted by ritonavir, an inhibitor of cytochrome P450 CYP3A4 to prolong activity (Shah et al., 2022). Paxlovid should however be initiated within 5 days of onset of symptoms in patients at risk of progressing to a severe state, but not to those requiring hospitalization due to severe COVID-19 (Komorowski et al., 2022) nor to those with severe hepatic impairment (Chaplin, 2022; Chen et al., 2023). Anti-inflammatory therapy includes timely administration of steroidal and nonsteroidal agents such as dexamethasone which, despite reducing mortality by ∼30% in ventilated patients, was associated with severe side effects (Noreen et al., 2021) and with worsened clinical outcomes (De Stefano et al., 2020; Noreen et al., 2021). Inhibitors of Janus kinase (JAK1 and 2), such as baricitinib, have been approved for use against SARS-CoV-2 in emergency situations in the European Union and the USA (Bellino, 2022; Huang et al., 2022; Rubin, 2022). The recovery collaborative group (2022) reported that baricitinib reduced mortality in severe COVID-19 by about one-fifth (Abani et al., 2022). The humanized monoclonal antibody against IL-6, tocilizumab has been approved in Canada, Europe, and the USA (Bellino, 2022; Canada, 2022; FDA, 2022; Mohseni Afshar et al., 2023). Despite elevation of IL-6 in the cytokine storm of SARS-CoV-2-infected patients, caution has been expressed on the use of anti-IL-6 therapy because conditions worsened in some patients (Montazersaheb et al., 2022). Moreover, IL-6 blockade may not be sufficient in critically ill COVID-19 patients and may increase the risks for severe infection (Montazersaheb et al., 2022). In addition, cell-based strategies have been proposed to mitigate the abundance of infection fighting neutrophils and neutrophil extracellular traps during the progressive pulmonary dysfunction of critically ill COVID-19 patients (Mozzini and Girelli, 2020; Narasaraju et al., 2020; Veras et al., 2023). New therapeutic avenues are needed to safeguard vulnerable populations against a persistent incidence of COVID-19 infection.

### Hexarelin attenuates lung cytokine levels and improves survival

Transgenic mice possessing the human ACE2 protein, which is expressed under regulation of the human cytokeratin-18 promoter in epithelial cells (K18-hACE2), were treated with a subcutaneous injection of hexarelin (10 μmol/kg) or 0.9% NaCl vehicle 30 min prior to intranasal infection with 250 TCID_50_ of SARS-CoV-2 (strain Delta, B.1.617.2, National Microbiology Laboratory, Winnipeg, Manitoba, Canada). Thereafter, mice were treated daily with 0.9% NaCl or hexarelin for 9 days. The experimental protocol was approved by the institutional animal care committee of Université Laval (CPAUL, 2020-594), in accordance with the guidelines for the care and use of laboratory animals of the Canadian Council on Animal Care and the US National Institute of Health.

**FIGURE 1:**
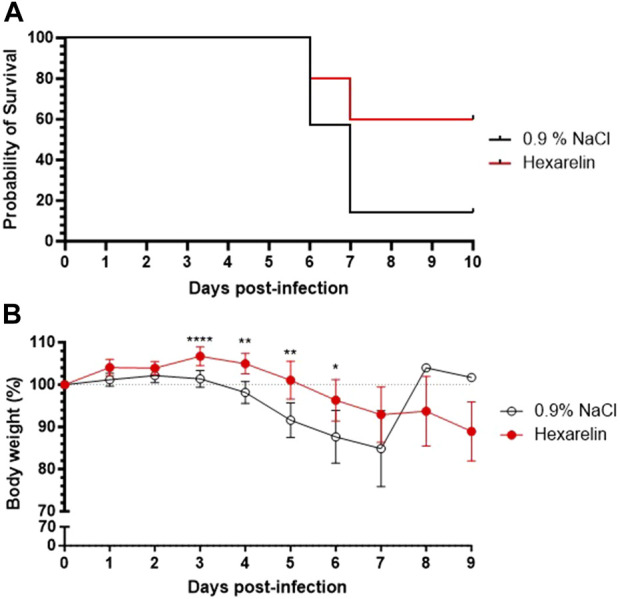
Treatment with hexarelin improves the survival of SARS-CoV-2-infected mice. **(A)** Kaplan Meier curve of K18-hACE2 mice infected with SARS-CoV-2. **(B)** Daily mean body weight of SARS-CoV-2-infected mice expressed as a percentage of body weight at day 0 (*n* = 7 mice per group).

Daily treatment with the CD36 ligand increased survival of the infected mice relative to vehicle-treated mice (Figure 1A). Hexarelin treatment also reduced the accompanied weight loss of infected mice relative to the placebo-treated group (Figure 1B). Moreover, lung homogenates from the hexarelin-treated infected mice exhibited decreased cytokine levels relative to placebo-treated mice: e.g., CCL2, CCL4, interferon (IFN) α and IFN γ, were significantly reduced by 44%, 59%, 43% and 35%, respectively (Table 1).

**TABLE 1:** Hexarelin decreased cytokine levels in lung homogenates at day 3 after SARS-CoV-2 infection.

Cytokines	Group (n = 3)	pg/mg ± SEM	*p* values
CCL2	0.9% NaCl	131 ± 5	0.0082*
	Hexarelin	73 ± 11	
CCL3	0.9% NaCl	6.9 ± 0.5	0.1066
	Hexarelin	5.0 ± 0.8	
CCL4	0.9% NaCl	12.1 ± 1.4	0.0084*
	Hexarelin	5.0 ± 0.5	
CXCL9	0.9% NaCl	431 ± 84	0.7000
	Hexarelin	383 ± 12	
GROα	0.9% NaCl	7.5 ± 0.8	0.0978
	Hexarelin	5.3 ± 0.7	
IFNα	0.9% NaCl	23 ± 1	0.0463*
	Hexarelin	13 ± 3	
IFNβ	0.9% NaCl	13 ± 2	0.2978
	Hexarelin	9 ± 2	
IFNγ	0.9% NaCl	1.7 ± 0.2	0.0477*
	Hexarelin	1.1 ± 0.1	
IL-1β	0.9% NaCl	1.5 ± 0.1	0.0698
	Hexarelin	1.2 ± 0.1	
IL-6	0.9% NaCl	32 ± 4	0.1632
	Hexarelin	24 ± 3	
IP-10	0.9% NaCl	96 ± 9	0.7891
	Hexarelin	91 ± 14	
RANTES	0.9% NaCl	167 ± 11	0.1413
	Hexarelin	144 ± 7	
TNFα	0.9% NaCl	7.5 ± 0.8	0.0978
	Hexarelin	5.3 ± 0.7	

## Discussion

The SARS-CoV-2 epidemic represents an unprecedented medical challenge worldwide (WHO, 2020). Disease course during SARS-CoV-2 infection features a similar profile to that of other highly pathogenic coronaviruses such as SARS-CoV and MERS-CoV, characterized by extensive leukocyte infiltration dominated by macrophages in post-mortem lungs of infected patients (Zhang et al., 2022). Mortality from SARS-CoV-2 has been mainly associated with the so-called cytokine storm creating a hyper-inflammatory state in infected patient lungs, leading to ARDS, compromised respiratory function, and multiple organ failure. The CD36 receptor has been associated with acute lung injury, contributing to the hyper-inflammatory response of lung macrophages after animal exposure to hydrogen peroxide, lipopolysaccharide, and malaria (Alghanim et al., 2022; Sun et al., 2022). As a co-receptor, CD36 regulates the assembly of TLR2/6 and TLR4/6 heterodimers for activating the transcription of pro-inflammatory cytokines, inflammasome priming, and production of reactive oxygen species in response to endogenous oxidized lipids (Sheedy et al., 2013; Mellal et al., 2019). Moreover, the E protein of SARS-CoV-2, which plays a central role in cytokine secretion and progression towards ARDS, was found to bind to CD36 and shown to be involved in COVID-19-induced thrombosis (Tang et al., 2023).

Hexarelin was previously shown to mitigate the development of HCl-induced ARDS-like symptoms in the bronchus and hypothesized to be able to attenuate lung inflammation and cytokine release in SARS-CoV-2-infected mice (Zambelli et al., 2021). We theorized that CD36 ligands, which modulate macrophage-driven inflammation, may curb the overreactive inflammatory response in a SARS-CoV-2-infected transgenic mouse model. We have found that the CD36 ligand hexarelin dampened many pro-inflammatory pathways, reducing type I interferon activity, chemokine levels and NLRP3 inflammasome priming. A limitation of our study is the unselective, dual binding of hexarelin towards CD36 and the ghrelin receptor (GHS-R1), both of which are expressed on macrophages. Future studies using selective azapeptide ligands are mandatory; nonetheless, the promise of modulating monocyte- and macrophage-driven hyper-cytokinemia by targeting CD36 suggests potential for mitigating SARS-CoV-2-mediated activation of inflammatory responses responsible for disease severity.

## Data Availability

The original contributions presented in the study are included in the article/supplementary material, further inquiries can be directed to the corresponding author.
